# Ashwagandha (*Withania somnifera*) in insulin resistance and metabolic syndrome: A literature review on mechanisms

**DOI:** 10.22038/ijbms.2025.86747.18741

**Published:** 2026

**Authors:** Emad Azimi, Maryam Rameshrad, Mahboobeh Ghasemzadeh Rahbardar, Hossein Hosseinzadeh

**Affiliations:** 1 Student Research Committee, Mashhad University of Medical Sciences, Mashhad, Iran; 2 Department of Pharmacodynamics and Toxicology, School of Pharmacy, Mashhad University of Medical Sciences, Mashhad, Iran; 3 Pharmaceutical Research Center, Pharmaceutical Technology Institute, Mashhad University of Medical Sciences, Mashhad, Iran

**Keywords:** Atherosclerosis, Dyslipidemia, Hypertension, Indian ginseng, Metabolic syndrome, Withania somnifera

## Abstract

Metabolic syndrome is characterized by obesity, insulin resistance, dyslipidemia, and hypertension. *Withania somnifer*a, commonly known as ashwagandha or Indian winter cherry, belongs to the *Solanaceae *family. *W. somnifera*, particularly its powdered root, is a fundamental component of traditional Indian medicine. *W. somnifera* (Ashwagandha) exhibits pharmacological activities, including immunomodulatory, anti-stress, and neuroprotective effects in animal models. Also, preclinical and clinical studies demonstrate its anti-inflammatory and antiviral properties. In rodent studies, ashwagandha regulates apoptosis and modulates reactive oxygen species (ROS) levels as well as mitochondrial activity. Additionally, it improves endothelial function in rats, dogs, and human brain endothelial cells. Research conducted in both living organisms and controlled laboratory conditions has demonstrated that *W. somnifera* alleviates the symptoms of metabolic syndrome. It positively affects diabetes by inhibiting dipeptidyl peptidase-4 (DPP-4), α-glucosidase, and α-amylase, while simultaneously increasing pancreatic insulin secretion and improving insulin sensitivity in organs. It has a vasodilatory and diuretic effect. Ashwagandha reduces the activity of 3-hydroxy-3-methylglutaryl coenzyme A (HMG-CoA) reductase. It modulates the gene expression of peroxisome proliferator-activated receptor (PPAR)-γ. It regulates the gene expression of sterol regulatory element-binding protein (SREBP)-1c and *CYP7A1*. It increases the secretion of bile acids that eliminate excess cholesterol. It reduces oxidative stress and inflammation while protecting the body from the harmful effects of elevated cholesterol. This study aims to compile a variety of research findings on the effectiveness of ashwagandha in managing metabolic syndrome.

## Introduction

Insulin resistance syndrome, also known as metabolic syndrome, is increasingly recognized as a significant global health challenge. This condition is characterized by a combination of abdominal obesity, insulin resistance, dyslipidemia, and hypertension, which may occur with impaired glucose tolerance, a pro-inflammatory state, and a pro-thrombotic condition (1, 2). Contributing factors to the development of insulin resistance syndrome include a sedentary lifestyle, rapid urbanization, and excessive caloric intake (3, 4). 

### Insulin resistance syndrome

Indeed, environmental factors and genetic predispositions play significant roles in the development of insulin resistance syndrome, which is characterized by an increase in adipose tissue mass. When subcutaneous adipose tissue reaches its capacity, free fatty acids (FFAs) can be transported to vital organs, particularly the liver. This process raises the risk of developing type 2 diabetes and atherogenic dyslipidemia by increasing insulin resistance in muscle tissue and enhancing gluconeogenesis and de novo lipogenesis in the liver. Additionally, in cases of fatty liver, a glucagon-resistant state affects amino acid metabolism. This resistance leads to the conversion of amino acids into glucose instead of their conversion into urea, creating a vicious cycle that can precede the onset of diabetes (5). Moreover, several factors contribute to obesity-induced sympathetic activation, including hyperleptinemia, hyperinsulinemia, visceral obesity, sleep apnea, elevated non-esterified fatty acids, disturbances in nitric oxide levels, suppressed anger, altered eicosanoid metabolism, increased cytokine levels, and high levels of active tri-iodothyronine. This sympathetic activation can lead to severe health issues such as sodium retention, increased cardiac output and heart rate, elevated vascular resistance, activation of the renin-angiotensin system, high blood pressure, enhanced oxygen consumption, sudden death, insulin resistance, increased non-esterified fatty acids, diabetes, dyslipidemia, and congestive heart failure (6). Addressing these critical health concerns is essential, as they represent the five key components of insulin resistance syndrome: hypertension, diabetes, obesity, dyslipidemia, and atherosclerosis. By understanding and managing these issues, we can significantly improve overall health and reduce the risk of serious complications. Insulin resistance syndrome is a cluster of dysregulations that requires multidrug therapy for effective management. Therefore, introducing drugs, compounds, or medicinal foods that address these multiple issues can enhance patient compliance. 

### Botanical medicine in insulin resistance syndrome

There has been a growing fascination with the utilization of botanical remedies to avert and manage a diverse array of ailments, notably cardiovascular conditions. This is primarily due to their commendable attributes, such as safety, effectiveness, cultural acceptance, and minimal adverse reactions (7). In addition to focusing on drug repositioning for managing complications of metabolic syndrome (8-12), several studies have also explored the potential effectiveness of various medicinal plants in addressing multiple issues related to metabolic syndrome. Notable examples of such plants include: *Tinospora cordifolia* (giloy) (13), *Ziziphus jujuba *(Jujube) (14), *Juglans regia* (walnut) (15), *Coriandrum sativum *(coriander) (16), *Portulaca oleracea* (purslane) (17), *Zingiber officinale* (ginger) (18), *Panax ginseng* (ginseng) (19), *Solanum melongena* (eggplant) (20), *Boswellia *species (21), *Abelmoschus esculentus* (okra) (22), *Ginkgo biloba *(23),* Crataegus pinnatifida *(Chinese hawthorn) (24), *Aloe vera *(25), *Silybum marianum* (milk thistle) (26), *Capsicum annuum *(27)*, Persea americana* (avocado) (28), *Garcinia mangostana *(29), *Berberis vulgaris *(30),* Rosmarinus officinalis *(rosemary) (31), *Vitis vinifera *(grapes) (32), *Allium sativum* (garlic) (33), and *Nigella sativa *(34). These plants offer probable therapeutic options for managing the complications associated with metabolic syndrome.

### Withania somnifera botanical characterization

*W. somnifera* is an evergreen, stout, shrubby plant that grows to a height of 1.5–2 m. It is a member of the *Solanaceae* family. It grows in the drier parts of tropical and subtropical zones of the world, including warmer parts of Europe, the Canary Islands, and tropical Africa to South Africa, Sri Lanka, the Middle East, China, and India. It is known as “Indian winter cherry”, “Indian ginseng”, “Ashwagandha”, “Asgandh”, “Punir”, and “Asgand” (35).

Indian ginseng is characterized by simple, shiny, smooth, and glabrous leaves that are ovate. The leaves are petiolate with a smooth margin, showing a dull green color and measuring 10 to 15 cm in length. The leaves are arranged alternately on vegetative shoots and oppositely on floral shoots. They have a bitter taste. The branches are round, straight, and can grow up to 60 to 120 cm long, covered in minute trichomes. Flowers appear solitary, are bisexual, and have a pedicel, measuring 4 to 6 cm in diameter, with colors ranging from greenish to yellow. The fruits are berry-like, small, and approximately 6 mm in diameter, characterized by an orange-red color. They contain tiny, smooth, and kidney-shaped seeds that are yellow and about 2.5 mm in diameter. The roots are stout and fleshy, tapering to a point, with a brownish-white or light-yellow color. They measure 15 to 25 cm in length and have a bitter taste with a horse-like smell (36, 37). 

**Figure 1:**
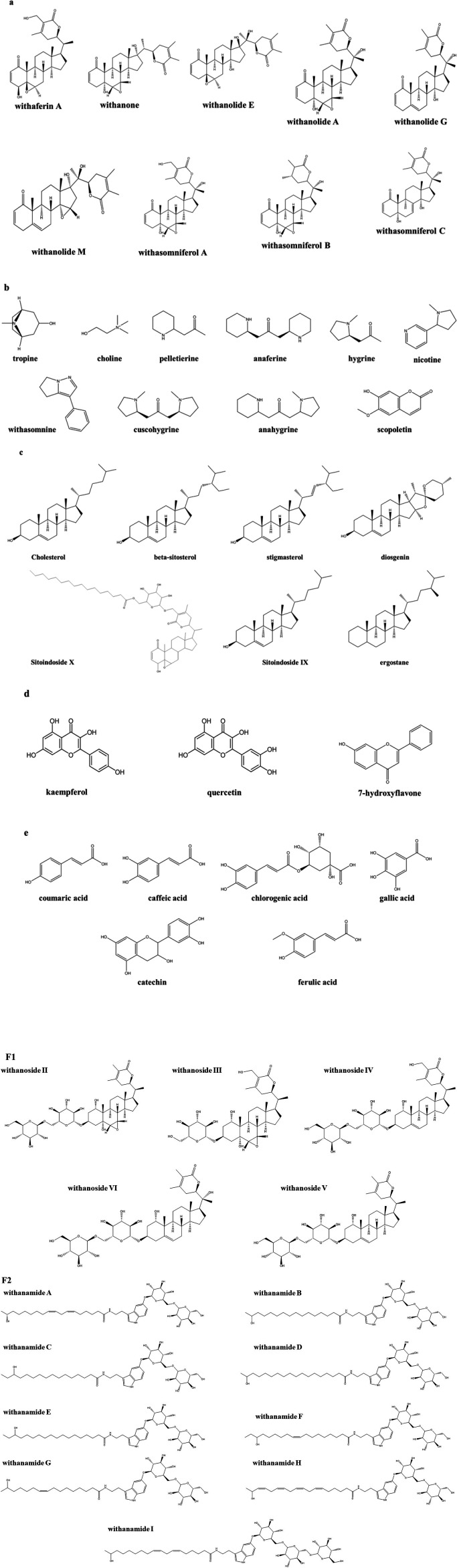
Some phytoactive constituents of Indian ginseng are presented

Characterization of the phytochemicals in *W. somnifera* revealed the presence of several classes of compounds, including steroidal lactones, alkaloids, steroids, flavonoids, phenolic compounds, glycosides, and other compounds (tannins, resin, fatty acids, organic acids, and amino acids) (35, 37, 38). Steroidal lactones and alkaloids are responsible for a wide range of pharmacological effects of Indian ginseng (39). The phytochemical structures of some compounds, based on information from PubChem, are presented in Figure 1. It is important to note that in a recently published article, hentriacontane was categorized as an alkaloid compound (37) despite being an alkane hydrocarbon. 

This characterization highlights the diverse range of bioactive compounds present in *W. somnifera* that are physiologically and pharmacologically active chemical components (40). This herb has been used for over 3,000 years as a medicinal herb in various medical systems, including Ayurvedic, Allopathic, Unani, and homeopathic medicine (37). Currently, there are more than ten marketed formulations of Indian ginseng (38). People refer to the specific aromatic young root of the plant as “ashwa” which means “horse” and “gandha” means fragrance (41), and have used it as an aphrodisiac, stimulant, tonic, narcotic, diuretic, and anthelmintic (42-44). Ashwagandha’s acylsterylglucosides and sitoindosides have anti-stress properties. An animal study has demonstrated that active components of ashwagandha, such as sitoindosides VIII and Withaferin-A, have intense anti-stress action against acute forms of experimental stress, including PTZ-induced defecation and urination, restraint stress-induced gastric ulcers, and forced swimming test (45). Some components in it promote immunomodulatory effects in mice and promote memory and learning in rats (46). This plant has been an essential component of Ayurvedic medicine that supports the nervous system (47). 

Preclinical studies have shown that Indian ginseng has therapeutic effects against hepatic (48) and neurological diseases (49), anxiety (50), and Parkinson’s disease (51). A review of both preclinical and clinical studies reported that* W. somnifera* may alleviate the symptoms of insomnia and depression, potentially through GABAergic and serotonergic pathways, and by modulating the hypothalamic-pituitary-adrenal axis and the sympathetic-adrenal-medullary system (52). Another review suggested that Indian ginseng inhibits the inflammasome proliferation of macrophages and oxidative stress and prevents acute liver injury in both preclinical and clinical models (53). Additionally, its role in managing COVID-19 has been explored in another review study. It can affect viral entry and load, immune homeostasis, and cytokine storms (54). Ko *et al*.’s comparable animal studies revealed significant gains in strength and muscular mass (55). According to a well-established clinical study by Wankhede *et al*. (56) and a review by Bonilla *et al*. (57), *W. somnifera* enhances body composition and encourages the retention of muscular mass by raising anabolic activity and lowering catabolic stress hormones.

### Withania somnifera safety

Hydroalcoholic extract of Indian ginseng has been reported to be safe at 2,000 mg/kg/day, orally, for 28 days in Wistar rats (58). In addition, prenatal exposure to 2,000 mg/kg/day of hydroalcoholic extract of Indian ginseng, orally, during days 5 to 19 of gestation in Wistar rats, did not show any maternal or developmental toxicity (59). The bacterial reverse mutation test indicated that Ashwagandha root extract, at concentrations ranging from 0.156 to 5.00 mg/plate, did not demonstrate any potential for mutagenicity or carcinogenicity. Additionally, a chromosome aberration assay using human peripheral blood lymphocytes exposed to the extract at concentrations of 0.25, 0.5, 1, and 2 mg/ml and *in vivo* micronucleus test in mammalian erythrocyte cells that received doses of 500, 1,000, and 2,000 mg/kg also showed that the extract had no inhibitory effects on the mitotic index, the formation of micronuclei, or cell proliferation, and did not induce chromosomal abnormalities. Furthermore, there were no signs of acute toxicity observed at a dose of 2,000 mg/kg when administered orally to female Wistar rats (60). Clinical studies did not report significant side effects related to Ashwagandha root extract at a dosage of 600 mg/day, taken orally, for up to 12 weeks (61, 62). 

In a recently published review, Quinones *et al*. reported that ashwagandha may have protective effects against stress-induced cortisol elevation and obesity (63). Further, Allah *et al*. highlighted the role of *W. somnifera* in metabolic health disorders such as hypertension, obesity, dyslipidemia, and diabetes (64). In the present study, we explore deeper into the botanical characteristics of *W. somnifera* and examine the mechanisms by which it impacts the physiological and biochemical components of insulin resistance syndrome; some mechanisms discussed in our study were not included in the work by Allah *et al*. We will explore these effects on diabetes, overweight, hypertension, dyslipidemia, and atherosclerosis in a structured manner, separating the analyses into *in vitro*, *in vivo*, and clinical studies. Additionally, we will include a section on poly herbal formulations that feature Indian ginseng as one of the constituents.

### Methodology

The databases of Web of Science, PubMed, and Google Scholar were searched for this narrative review by the following keywords: hypertension, “blood pressure”, hypotensive, antihypertensive, hypertensive, diuretic, diabetes, hyperglycemia, insulin, hypoglycemic, antihyperglycemic, antidiabetic, “blood glucose”, HbA1c, dyslipidemia, hyperlipidemia, “high cholesterol”, “high triglyceride”, hypercholesterolemia, hypertriglyceridemia, hypoglycemic, hypolipidemic, hypoglycaemic, hypolipidaemic, “lipid-lowering”, atherogenic, atherosclerosis, obesity, overweight, appetite, anti-obesity, “weight loss”, “bodyweight”, “food intake”, “feed intake”, “metabolic syndrome”, “metabolic syndromes”, “metabolic syndrome X”, “insulin resistance syndrome”, “dysmetabolic syndrome X”, “reaven syndrome X”, “metabolic cardiovascular syndrome”, “cardiometabolic syndrome”, “cardiometabolic syndromes”, “*Withania somnifera*”, “winter cherry”, “Indian ginseng”, ashwagandha, withanias, withaferins, withanolides and withanosides.

**Figure 2:**
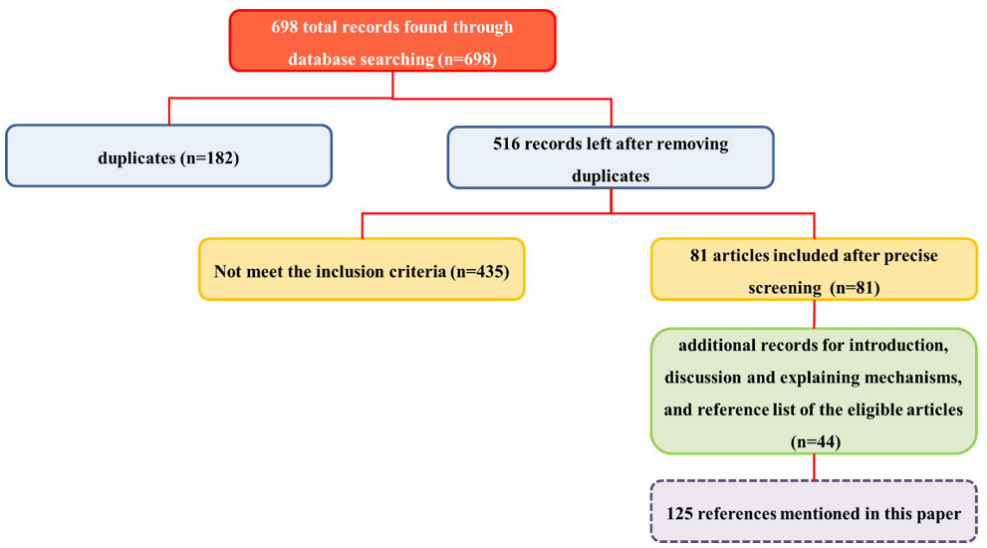
Schematic diagram showing the selection method and the number of included articles in brief

We expanded our search to include the reference lists of related articles to uncover additional findings. We considered all relevant *in vitro*, *in vivo*, and clinical studies published up to 2025, without restrictions on age or type of animal or cell line. Our primary outcome was the effects of Indian ginseng on diabetes, overweight, hypertension, dyslipidemia, and atherosclerosis. The mechanism by which Indian ginseng influenced these outcomes was considered a secondary outcome. We excluded narrative and systematic reviews, meta-analyses, opinions, and editorials. The titles and abstracts of the articles were reviewed for eligibility. Out of the 698 articles collected, we selected the relevant ones for inclusion (Figure 2). The results are categorized into three main sections: diabetes and overweight, hypertension, dyslipidemia, and atherosclerosis, each accompanied by a brief discussion and conclusion. Additionally, studies on poly herbal formulations that contain Indian ginseng or its constituents are included. 

## Results and Discussion

### The effects of Indian ginseng or its constituents on diabetes, overweight, and related complications

Diabetes is a key component of insulin resistance syndrome (65). It is associated with several complications, such as hypertension, atherosclerosis, and microcirculatory disorders, all of which contribute to increased morbidity and mortality rates (27). This section examines the mechanisms by which *W. somnifera* affects diabetes, obesity, and their related complications through *in vitro*, *in vivo*, and clinical studies (Table 1). 

### In silico studies

The results of *in silico* studies indicate the potential of withanolide A to act as an α-amylase and α-glucosidase inhibitor, contributing to diabetes management (66).

Furthermore, catechin, a flavonoid found in the methanolic extract of *W. somnifera* roots, exhibits strong antidiabetic properties. According to docking studies, catechin binds to and inhibits dipeptidyl peptidase-4 (DPP-4) (67).

### In vitro studies

In cultures of rat myotubes and adipocytes, both leaf and root extracts of *W. somnifera* increased glucose absorption in a dose-dependent manner, with the leaf extract showing greater activity than the root extract. Additionally, in pancreatic RIN-5 β-cells, the leaf extract enhanced insulin secretion at baseline levels (68). RIN-5F is a rat insulinoma cell line (69). 

The antidiabetic activity of the extract may be attributed to its phenolic and flavonoid compounds. Withaferin-A, derived from *W. somnifera*, exhibits anti-inflammatory effects and protects Langerhans cells from cytokine-induced damage (70). 

Babu *et al*. proposed that *W. somnifera* is effective in preventing pathogenesis induced by glycation. High levels of glucose can glycate collagen, leading to the formation of cross-links. The research revealed that the effects of the ethanolic extract of *W. somnifera* are comparable to those of metformin (71). 

It has been shown that the hydromethanolic leaf extract of Indian ginseng induces lipolytic effects and enhances glucose uptake in 3T3-L1 cells. Balkrishna *et al*.’s research offers mechanistic data supporting the activation of the AMPK/p38MAPK/ERK signaling pathway as an underlying mechanism driving the anti-adipogenic actions of *W. somnifera* leaf extract. Furthermore, supporting the possibility of using *W. somnifera* leaf as a successful treatment approach for obesity prevention are the results of their research (72). 3T3-L1 cells are a preadipocyte cell line that is developed from mouse embryo fibroblasts (73).

Additionally, leaf extracts of *W. somnifera* that are combined with ZnO nanoparticles have been shown to inhibit the activity of the enzymes α-amylase and α-glucosidase, contributing to their antidiabetic potential (74).

*Animal studies*

*W. somnifera* root extract is effective in reducing oxidative stress and mitochondrial dysfunction in the hypothalamus of diabetic rats. The hypothalamus is a crucial area of the brain that plays a key role in sensing glucose levels. In diabetic patients, oxidative stress can impair the hypothalamus’s function, leading to significant disruptions in neuronal metabolic functions. The root extract of *W. somnifera* not only improved mitochondrial function but also prevented oxidative damage in the hypothalamus of diabetic rats. Overall, *W. somnifera* root extract helps mitigate oxidative stress and mitochondrial dysfunction in this region of the brain (75).

In cases of alloxan-induced diabetes, both *W. somnifera* root extract and glibenclamide have been found to normalize glucose levels. This effect may be attributed to the plant’s phenolic compounds and anti-oxidant properties. Additionally, *W. somnifera* root extract, *W. somnifera* leaf extract, and glibenclamide led to a moderate increase in islet size and a significant reduction in pancreatic injuries (76). The regeneration and revitalization of β-cells could lead to increased production and release of insulin (77, 78). 

Research has shown that the extract of *W. somnifera* has a significant hypoglycemic effect in streptozotocin-induced diabetic rats (79, 80). It is suggested that *W. somnifera* regulates the changes in gonadal hormones caused by diabetes (81). Furthermore, *W. somnifera* protects against diabetes-induced testicular dysfunction in prepubertal rats by regulating thiol/glutathione (GSH) status and improving blood glucose levels (82). Studies indicate that anti-oxidants may be effective in treating infertility (83-85) and neuronal damage (86-88) associated with diabetes by balancing oxidant and pro-oxidant levels. In cases of alloxan-induced diabetes, ashwagandha root extract has been found to alleviate brain oxidative stress and depression (86). In non-insulin-dependent diabetic rats, *W. somnifera* significantly improves the insulin sensitivity index (89). Udayakumar *et al*. hypothesized that both *W. somnifera* leaf and root extracts may increase pancreatic insulin secretion. In diabetic rats, the effects of *W. somnifera* root extract and leaf extract on blood glucose levels were more pronounced than those of glibenclamide (90). 

Jena *et al*. demonstrated that the methanolic root extract of *W. somnifera* exhibits potent antihyperglycemic activity comparable to glibenclamide in streptozotocin-induced diabetic rats (91).

In diabetic mice, *W. somnifera* has been shown to protect against stress-induced hyperglycemia and hypercortisolemia (92). 

Furthermore, Tekula *et al*. confirmed that Withaferin-A effectively regulates type 1 diabetes in rats by modulating Nrf2/NFκB signaling pathways. This treatment significantly reduced levels of pro-inflammatory cytokines such as TNF-α and IL-6, decreased DNA fragmentation and apoptosis, and mitigated oxidative and nitrosative stress caused by streptozotocin (93). According to Lee *et al*. (2016), Withaferin-A has a remarkable ability to sensitize leptin, suggesting its potential as a treatment for metabolic syndrome associated with obesity and type 2 diabetes. The Lee *et al*. study demonstrates that Withaferin A reduced body weight, food intake, and fat mass in diet-induced obesity in mice, with minimal effects in ob/ob and db/db mice, indicating leptin-dependent effects. Withaferin A enhanced leptin potency, reduced hypothalamic endoplasmic reticulum stress, lowered PERKᵀʰʳ^980^ phosphorylation, and maintained energy expenditure in diet-induced obesity mice. It improved glucose homeostasis in diet-induced obesity, ob/ob, and db/db mice, with more substantial antidiabetic effects than celastrol, a highly potent leptin sensitizer, in ob/ob and db/db mice (94).

*W. somnifera* supplementation also reduces lipid peroxidation and significantly increases glutathione (GSH) levels in the pancreas (95). Additionally, the root extract containing Withaferin-A inhibits DPP-4 activity and, improves insulin resistance and β-cell dysfunction in type 2 diabetic rats (96). 

In studies examining high-fat diet-induced obesity in mice, the root water extract of *W. somnifera *has been shown to have no beneficial effects. It enhances the hepatic expression of the peroxisome proliferator-activated receptor (PPAR)-γ gene (97), which is known to play a positive role in the development of hepatic steatosis (98, 99), while also positively influencing pancreatic function (100). Kumari *et al*.’s findings (97) regarding the impact of *W. somnifera* root water extract on PPAR-γ expression contrast with those of Balkrishna *et al*. (72) and Abu Bakar *et al*.’s studies (101). The study by Balkrishna *et al*. reported a decrease in PPAR-γ expression. It demonstrated the anti-adipogenic effects of a hydromethanolic leaf extract of *W. somnifera* in 3T3-L1 adipocytes (72). Abu Bakar *et al*. showed that administration of Withaferin-A, a natural steroidal lactone found in *W. somnifera*, improved insulin sensitivity and glucose tolerance in mice made obese through a high-fat diet. Furthermore, Withaferin-A enhanced liver oxidative function and reduced levels of pro-inflammatory cytokines. It also decreased the expression of genes involved in glucose and lipid metabolism, including glucokinase (GCK), phosphoenolpyruvate carboxykinase (PCK1), PPARs, CD36, carnitine palmitoyltransferase 1 (CPT1), and fatty acid synthase (FAS) mRNA in the liver. Overall, Withaferin-A offers protection against obesity by reducing inflammation, oxidative stress, and insulin resistance (101). It is important to note that the explanation of Abu Bakar *et al*.’s findings in the article differed from what was presented in the abstract. Based on the results discussed in the text, we conclude the items mentioned above (101). The decrease in expression of GCK, PCK1, PPARs (PPARγ, PPARα), CD36, CPT1, and FAS in the liver, as reported in Abu Bakar *et al*. (2018), refers specifically to transcription (mRNA) levels, as measured by qRT-PCR. The study did not assess protein levels, and thus, it is not known whether these mRNA reductions correspond to decreased protein expression or activity. The authors explicitly note this as a limitation, suggesting that future studies should investigate protein-level changes to confirm the full mechanistic impact of Withaferin-A (101).

This highlights the different roles of PPAR-γ in various tissues (98) and the diverse effects of other types of *W. somnifera* extracts.

Dianex, a polyhedral formulation containing *W. somnifera*, has demonstrated hypoglycemic effects in both normal and diabetic mice, both in acute and long-term settings. Dianex is a polyherbal formulation made from the aqueous extracts of *Gymnema sylvestre*, *Eugenia jambolana*, *Momordica charantia*, *Azadirachta indica*, *Cassia auriculata*, *Aegle marmelos*, *W. somnifera*, and *Curcuma longa* (102).

*Clinical studies*

In a study involving human subjects with mild non-insulin-dependent diabetes mellitus and another group with mild hypercholesterolemia, the administration of *W. somnifera* root powder (1,000 mg three times a day for 30 days) resulted in significant hypoglycemic and hypocholesterolemic effects. Additionally, the extract demonstrated diuretic properties (103).

**Figure 3:**
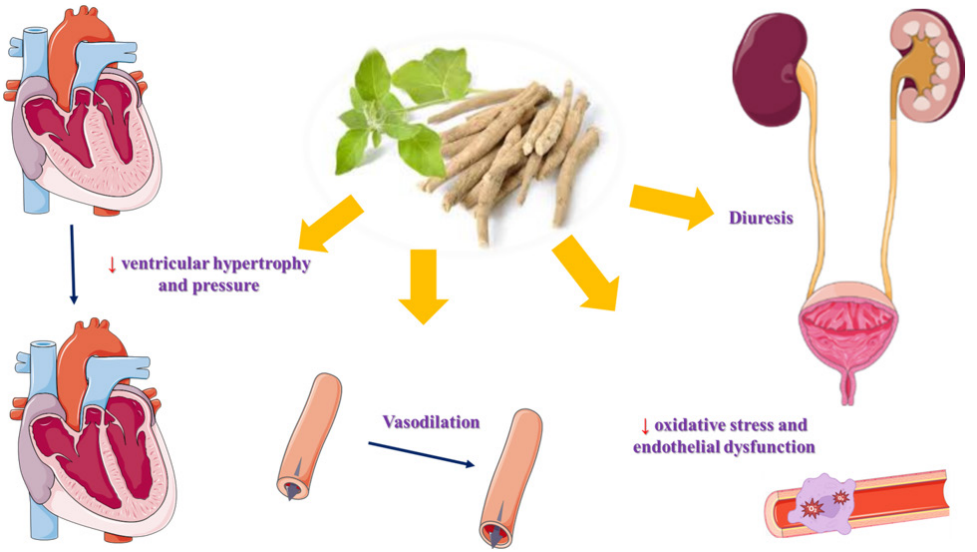
Antidiabetic mechanisms of *Withania somnifera *and its main active constituents

Beyond its anti-oxidant effects, *W. somnifera* exhibits two primary functions relevant to its antidiabetic effects: DPP-4 inhibition and the blocking of polysaccharide conversion into glucose. Furthermore, *W. somnifera* boosts the pancreatic secretion of insulin from existing β-cells within the islets of Langerhans. This process facilitates an increase in peripheral glucose consumption through various enzymatic pathways (Figure 3). 

### The effects of Indian ginseng or its constituents on hypertension

High blood pressure, also known as hypertension, can be detrimental to human health in various ways. It may lead to severe damage to vital organs, including the heart, brain, kidneys, and eyes (104).

*Animal studies*

In a study involving dogs, it has been demonstrated that *W. somnifera* extract reduces the hypotensive effects of acetylcholine while extending the hypertensive effects of adrenaline. Additionally, it exhibits hypotensive effects in normotensive animals (105). 

In both preventive and therapeutic settings, *W. somnifera* therapy has been shown to reduce right ventricular hypertrophy and pressure. In the preventive context, it decreases inflammation, oxidative stress, and endothelial dysfunction. Additionally, it reverses the remodeling of the pulmonary vascular system associated with pulmonary hypertension. *W. somnifera *exhibits anti-inflammatory, pro-apoptotic, vasodilatory, and anti-oxidant properties, making it a valuable option for managing pulmonary hypertension. High-performance liquid chromatography (HPLC) investigations have identified several bioactive compounds, primarily withaferin A and withanolide A, which are likely responsible for the protective effects of *W. somnifera* (106). Withaferin A enhances the activity of endogenous anti-oxidant enzymes such as superoxide dismutase while simultaneously reducing lipid peroxidation, giving *W. somnifera* its strong anti-oxidant capabilities (107). 

*Clinical trials*

In a double-blind, placebo-controlled, randomized crossover study, *W. somnifera* extract (500 mg of dried aqueous extract of roots and leaves, taken twice daily) showed a statistically significant reduction in aortic pressure among normotensive volunteers experiencing mental stress (108). 

**Figure 4:**
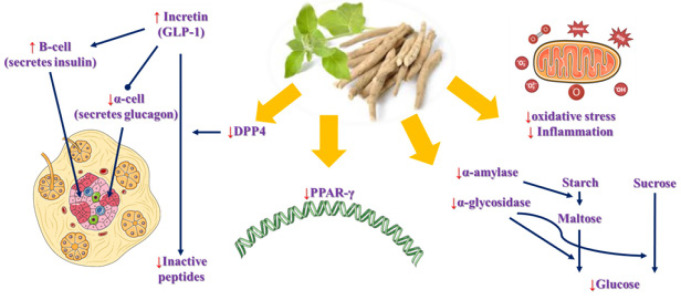
Antihypertensive mechanisms of *Withania somnifera* and its main active constituents

Several mechanisms seem to contribute to the antihypertensive effects of *W. somnifera*, particularly its diuretic properties, as well as its modulation of inflammation and oxidative stress (Figure 4 and Table 2).

### The effects of Indian ginseng or its constituents on dyslipidemia and atherosclerosis

Dyslipidemia and obesity are components of insulin resistance syndrome (109). Dyslipemia is considered one of the main predisposing factors for diabetes and cardiovascular diseases (110). The deposit of lipids in vessel walls aggravates atherosclerosis (111). This part discusses the most relevant articles that evaluated the role of *W. somnifera* in dyslipidemia, atherosclerosis, and related complications (Table 2).

*In vitro studies*

Soh *et al*. examined the effects of withanolide-A on damage caused by 7-ketocholesterol (7KC), a cholesterol oxidation product, in human brain endothelial cells (hCMEC/D3). Elevated levels of 7KC are found in the plasma of individuals with hypercholesterolemia or diabetes mellitus and are also present in atherosclerotic plaques. When hCMEC/D3 cells are treated with 7KC, withanolide-A promotes cell survival, reduces COX-2 activity and the expression of inflammatory genes, and decreases the levels of reactive oxygen species. Furthermore, withanolide-A mitigates the increases in thrombin activity and clotting factor expression induced by 7KC, which are the final steps in the clotting cascade (112).

The findings of some experiments indicate that the withanolides exhibit anti-adipogenic properties. They demonstrated that compounds 1-6, including Withasilolide F, E, and Withasomniferol B (4-6), along with three novel withanolides, Withasilolides G, H, and I (1-3), significantly prevented adipogenesis and reduced the expansion of lipid droplets in 3T3-L1 cells. Moreover, the mRNA expression levels of Fabp4 and Adipsin, which are adipocyte marker genes, were significantly diminished after treatment with 25 µM of these compounds. Treatment with compounds 1-5 raised the mRNA expression of HSL and ATGL, the lipolytic genes, in 3T3-L1 cells; however, compound 6 did not up-regulate the mRNA expression of ATGL. Conversely, the mRNA expression of SREBP1, the lipogenic gene, was down-regulated after treatment with 25 µM of compounds 1-6 during adipogenesis. The studies indicated that compounds 1-6 may enhance lipid metabolism by increasing lipolysis and suppressing lipogenesis (113).

*Animal studies*

Dietary supplementation with *W. somnifera* root powder significantly reduced the levels of cholesterol, triglycerides, and total lipid levels in the egg yolk of birds (114, 115).

In diabetic rats treated with *W. somnifera*, elevated levels of lipid peroxidation (LPO) were significantly reduced. In contrast, the levels of anti-oxidant enzymes increased. This suggests that *W. somnifera* supplements help reduce oxidative stress and hyperlipidemia, while also protecting the heart from injury. Additionally, these supplements may help prevent diabetes-related complications (116). Tiwari *et al*. showed protective effects of *W. somnifera *against diabetes-induced dyslipidemia in rats. LDL and total cholesterol levels decreased after taking *W. somnifera*; these decreases were seen at all oral doses (250, 500, and 1000 mg/kg/day, for 28 days). *W. somnifera* therapy, however, did not restore TG levels to the same degree as LDL and total cholesterol, indicating a specific effect on lipid metabolism (117). In the other study by the Tiwari group, treatment of diabetic rats with *W. somnifera* resulted in less weight loss (118), which could be because of its ability to preserve muscle mass and lower oxidative stress (56, 57)

In alloxan-induced diabetic rats, extracts from both the root and leaf of *W. somnifera* demonstrated antidiabetic and antihyperlipidaemic properties. These extracts were effective in lowering the blood levels of cholesterol, triglycerides, and phospholipids, while simultaneously increasing HDL levels (90).

Acid phosphatase levels and activity are elevated in diabetic rats due to changes in cell membrane structure caused by diabetes and hyperlipidemia. However, these damages can be reversed in groups treated with *W. somnifera*. The administration of root and leaf extracts of *W. somnifera*, along with glibenclamide, may help reduce acid phosphatase levels and activity. This study conclusively demonstrates that the root and leaf extracts of *W. somnifera* exhibit hypoglycemic and hypolipidemic properties in rats with alloxan-induced diabetes mellitus (90).

One potential mechanism for the cholesterol-lowering effects of *W. somnifera* could be a reduction in the activity of the enzyme 3-hydroxy-3-methylglutaryl coenzyme A (HMG-CoA) reductase, along with an increase in the excretion of bile acids and cholesterol through fecal sterols. The hypocholesterolemic action of *W. somnifera* may be mediated by enhanced production of bile acids, which facilitate the removal of excess body cholesterol. Mice given *W. somnifera* root powder exhibited improved hepatic anti-oxidant activities, suggesting that the root’s fiber, phytosterols, polyphenols, flavonoids, and vitamin C may contribute to alleviating hyperlipidemic conditions (119). In rabbits with high-cholesterol diet-induced dyslipidemia, *W. somnifera* also improved anti-oxidant activities and reduced dyslipidemia (120). 

Withaferin-A (0.75 or 1.5 mg/kg (dose interval indeterminate), orally for 7 days) showed anti-obesity effects in mice fed on a high-fat diet. It enhances energy expenditure through thermogenic gene expression and adipose tissue browning without affecting food intake. Furthermore, it down-regulates mRNA expression of lipogenesis-related genes (e.g., SREBP1 and FAS) in the liver and adipogenesis-related genes (e.g., PPARγ and C/EBPα) in epididymal fat, decreasing hepatic and adipose lipid storage. withaferin-A administration up-regulates thermogenic genes (e.g., Ucp1, Pgc1α, Nrf1, and Prdm16) in brown adipose tissue and enhances thermogenesis. Withaferin A stimulates AMP-activated protein kinase (AMPK) phosphorylation at Thr172 in BAT, promoting catabolic processes like fatty acid oxidation and inhibiting anabolic pathways. It can enhance mitochondrial biogenesis. *W. somnifera* increases phosphorylation of p38 and ERK1/2 mitogen-activated protein kinases (MAPKs) in brown adipose tissue and subcutaneous white adipose tissue. These pathways up-regulate UCP1 and PGC-1α transcription, critical for thermogenesis and mitochondrial biogenesis. *In vitro*, withaferin-A-treated 3T3-L1 adipocytes show dose-dependent UCP1 and PGC-1α expression, which is suppressed by p38 (SB203580) or ERK (U0126) inhibitors, confirming pathway dependence. Withaferin-A ‘s anti-obesity effects are driven by increased energy expenditure via AMPK activation in brown adipose tissue, p38 and ERK1/2 MAPK signaling in brown adipose tissue and subcutaneous white adipose tissue, and subcutaneous white adipose tissue browning. These mechanisms enhance thermogenic gene expression and mitochondrial biogenesis while reducing lipid accumulation, highlighting withaferin-A’s therapeutic potential for obesity management (121).

*W. somnifera* increases the mRNA expression of lipid metabolism-related genes, including sterol regulatory element-binding protein 1c (SREBP-1c) and cytochrome P450 7A1 (CYP7A1), in the liver of HFD-fed mice (97). However, in an *in vitro* study, hydromethanolic leaf extract of *W. somnifera *decreased the formation of lipid droplets and accumulation of intracellular lipids in 3T3-L1 adipocytes, accompanied by a decrease in the expression of the SREBP1c gene (72). CYP7A1 plays a critical role in bile acid synthesis (122), while SREBP-1C enhances the uptake of circulating LDL by hepatocytes (123). These findings highlight tissue-specific actions of *W. somnifera*, promoting lipid catabolism in the liver while inhibiting adipogenesis in adipose tissue. It should be noted that the effect of SREBP on the lipid profile could be complex. Its activation may lead to an overexpression of hepatic LDL receptors, enhancing the uptake of circulating LDL by hepatocytes. However, it could also increase hepatic triglyceride and cholesterol synthesis, thereby elevating blood cholesterol levels (123). 

Specific polyherbal formulations containing Indian ginseng have shown positive effects in treating dyslipidemia. The herbal composition known as Caps HT2 includes various plants parts that have been extracted using methanol, such as *Commiphora mukul, Allium sativum, Plumbago indica, Semecarpus anacardium, Hemidesmus indicus, Terminalia arjuna, Tinospora cordifolia, Ocimum sanctum, and W. somnifera*. When this formulation was administered to rats with diet-induced hyperlipidemia for 30 days, it demonstrated a significant hypolipidemic effect. Notably, the administration of Caps HT2 markedly increased HDL cholesterol levels (124).

Ambrex is a polyherbal formulation that contains *W. somnifera*, *Orchis mascula, Cycas circinalis, Shorea robusta*, and amber. It reduces total cholesterol and triglycerides in male Wistar rats (125). 

**Figure 5:**
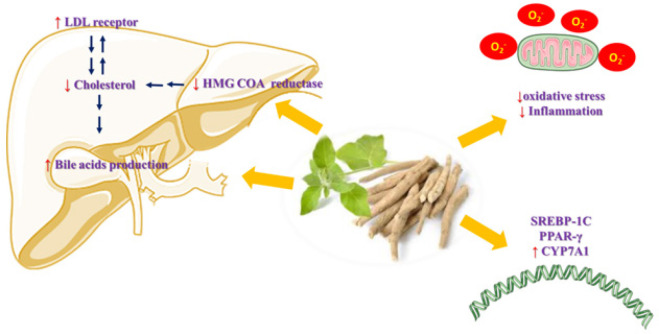
This figure illustrates the antihyperlipidemic and antiatherosclerotic mechanisms of *Withania somnifera*, commonly known as Indian ginseng, and its main active constituents

The antihyperlipidemic effects of *W. somnifera* are multifaceted. Primarily, these effects are related to the mobilization of low-density lipoprotein cholesterol (LDL-C) from the blood to the liver, where it is used for bile acid synthesis, thereby eliminating excess cholesterol. Additionally, *W. somnifera* reduces the activity of Hydroxymethylglutaryl-CoA (HMG-CoA) reductase and lowers inflammation, oxidative stress, and thrombin activity, all of which contribute to its antiatherosclerotic properties (Figure 5).

### Conclusion and future direction

Oxidative damage is linked to the development of hypertension, dyslipidemia, atherosclerosis, and complications related to diabetes. Patients with insulin resistance syndrome experience chronic oxidative stress, which arises from an imbalance between the production of free radicals and the body’s ability to neutralize them. Indian ginseng has been shown to restore cellular defense mechanisms, prevent lipid peroxidation, and protect tissues from oxidative injuries. 

Additionally, Indian ginseng offers significant antidiabetic benefits by inhibiting DPP-4 and blocking the conversion of polysaccharides into glucose. It enhances the secretion of insulin from the pancreas and improves peripheral glucose metabolism. 

Beyond its vasodilatory effects, Indian ginseng promotes diuresis. It supports the expression of genes related to lipid and glucose metabolism, while also reducing inflammation and thrombosis. It increases the excretion of bile acids and cholesterol through fecal sterol excretion. It possesses anti-adipogenic action and promotes the browning of subcutaneous fat by activating the AMPK/p38 MAPK/ERK signaling pathways.

*W. somnifera* and its primary constituents have various effects on PPAR-γ, with most of them exhibiting inhibitory effects on the expression of this gene in 3T3-L1 adipocytes and the liver tissue of high-fat-induced obesity. These effects are related to the different roles of PPAR-γ in various tissues, as well as the varied impacts of other types of *W. somnifera* extracts.

**Figure 6:**
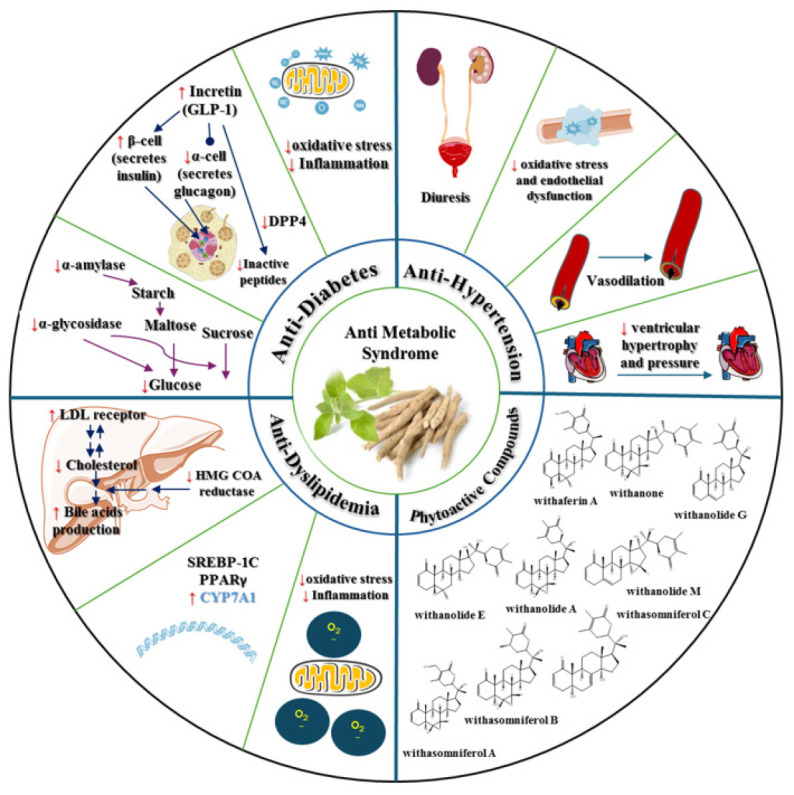
Some phytoactive constituents of Indian ginseng and their mechanistic impacts on metabolic syndrome components, including hypertension, diabetes, and dyslipidemia

These numerous properties (Figure 6) make Indian ginseng an ideal multi-purpose compound for patients with diabetes mellitus, hypertension, dyslipidemia, cardiovascular diseases, and metabolic syndrome. However, these findings need to be verified through extensive clinical trials. More research is required to better understand the potential side effects and to determine the optimal dosage of this plant. Additionally, the impact of Indian ginseng and the duration of its use in individuals with insulin resistance syndrome should be explored further. Moreover, various factors, including the timing of plant harvesting, geographical differences in cultivation, and the type of extract, can impact secondary metabolites and pharmacological effects.

A: Steroidal lactones (withaferin A, withanone, withanolide (A, E, G, M) (35, 37, 38), withasomniferols A-C (37)) B: Alkaloids (nicotine (35, 37, 38), tropine, choline, pelletierine, anaferine, cuscohygrine (37, 38), hygrine, withasomnine, anahygrine (37), scopoletin (38)) C: Steroids (cholesterol, β-sitosterol, stigmasterol, diosgenin, sitoindosides (IX, X) (35, 37, 38), and ergostane (37)) D: Flavonoids (kaempferol, quercetin (35, 37, 38), and 7-hydroxyflavone (37)) E: Phenolic compounds (coumaric acid, caffeic acid, chlorogenic acid, gallic acid, catechin, and ferulic acid (37)) F1 & F2: Glycosides (withanosides II-VI, withanamides A-I (37))

**Table 1:** Effects of Indian ginseng or its constituents on diabetes, overweight, and related complications

Indian ginseng or its constituents	Experimental model	Results	Ref.
Hydromethanolic leaf extract of *Withania somnifera* (1-30 μg/ml) for 24 hr	3T3-L1 preadipocytes	↓ In the formation of lipid droplets in 3T3-L1 adipocytes ↓ Intracellular lipid accumulation ↓ mRNA expression of C/EBPα, C/EBPβ, PPARγ, FAS, DGAT1, PLIN2, SREBP1c) ↓Pprotein levels of both C/EBPβ and PPARγ ↑ ATGL, HSL, GLT4 mRNA expression ↑TFAM, Cyt C, TMEM26, FGF21, CPT1, PGC1 α, UCP1, PRDM 16 mRNA expression ↑ p-AMPK, p-p38MAPK, p-ERK, PGC1α	(72)
Hydromethanolic leaf extract of *W. somnifera* 3, 10, and 30 μg/ml for 5 days	*C. elegans*, strains N2 (wild type)	↓Fat deposition ↓Fat-5, fat-6, fat-7	
Withaferin-A (1.25 mg/kg/day), 12 weeks, orally	High-fat diet (for 12 weeks) induced Obesity in C57BL/6J mice, accompanied by the Withaferin-A therapy	↓Body weight ↓Energy intake ↓Food intake ↑Water intake ↓Liver index ↑Glycogen content ↑Serum adiponectin ↓Serum leptin ↓Serum total cholesterol ↓Serum-free fatty acid ↓Insulin Resistance ↓Hepatic steatosis ↓Oxidative stress ↓Inflammation ↓Hepatic expression of PPARγ, PPARα, CD36, FAS, CPT1, GCK, and PCK1 No improving effect on the hepatic expression of LXRα and SREBP-1c	(101)
Withaferin A (10 μM)	L6 skeletal myoblasts and 3T3 adipocytes to evaluate glucose uptake	↑Glucose uptake in rat myotubes and adipocytes	(68)
Leaf and root methanol extracts of *W. somnifera* (100 µ/ml)	Pancreatic RIN-5F to evaluate insulin secretion	↑Insulin secretion in basal pancreatic β-cells (with leaf but not root extract)	
Root and leaf ethanol extract of *W. somnifera* (100 & 200 mg/kg/day), orally, for 8 weeks	Alloxan-induced diabetes mellitus in male Wistar rats	↓Blood glucose ↓Urine glucose ↓Glucose-6-phosphatase ↓ Pancreas injuries ↑Liver glycogen ↑Superoxide dismutase ↑Catalase ↑Glutathione peroxidase ↑Reduced glutathione ↑Glutathione-S-transferase ↑Vitamin C plasma level ↑Vitamin E plasma level	(76)
Ethanolic root extract of *W. somnifera* (250, 500, and 1000 mg/kg/day), orally, for 28 days	Nicotinamide and streptozotocin-induced diabetes in Wistar rats (sex indeterminate)	↓ Blood glucose levels ↑ Insulin secretion	(80)
Ethanolic root extract of *W. somnifera *(250, 500, and 1000 mg/kg/day), orally, for 28 days	Nicotinamide and streptozotocin-induced diabetes in Wistar rats (both sexes)	↑ Body weight	(118)
*W. somnifera* aqueous root extract (250, 500, and 1000 mg/kg/day), orally, for 15 days	streptozotocin-induced diabetes-induced Testicular oxidative impairments in prepubertal male CFT-Wistar rats	↑Body weight ↑Testis to body weight ↓Blood glucose ↓Lipid peroxidation ↑Testis glucose 6-phosphate dehydrogenase ↑Testis lactate dehydrogenase ↑Testis catalase ↑Testis dehydrogenase (3β-HSD)	(82)
*W. somnifera* root extract (100 and 200 mg/kg/day), orally, for six weeks (type of extract indeterminate)	Alloxan-induced diabetes in male albino rats, six weeks before treatment with *W. somnifera* extract, Forced swim test to evaluate diabetes-induced depression	↓Immobility duration in the forced swim test ↑Serum serotonin ↓Brain total oxidative capacity ↑Brain total anti-oxidative capacity ↓Brain oxidative stress index ↓Brain malondialdehyde ↑Brain superoxide dismutase ↑Brain catalase ↑Brain reduced glutathione ↑Organization and regular arrangement of gray matter and cerebellum sections in histopathology	(86)
Aqueous root extract of *W. somnifera *(200 and 400 mg/kg/day) orally, for 5 weeks	Streptozotocin-induced diabetes in 2-day-old Wistar rat pups (sex indeterminate), 19 days before *W. somnifera *therapy	↓Blood glucose ↓HbA1c ↓Glycosylated hemoglobin ↓Insulin level ↑Insulin sensitivity index ↓Homeostasis model assessment of insulin resistance	(89)
*W. somnifera* root and leaf ethanol extracts, 100 and 200 mg/kg/day, orally, for 8 weeks	Alloxan-induced diabetes in male Wistar rats, 2 weeks before receiving *W. somnifera*	↓Blood glucose ↓HbA1C ↑Liver glycogen ↓Total cholesterol in the heart, liver, and kidneys ↓TG in the heart, liver, and kidney ↓Phospholipid in heart, liver, and kidney ↓Liver glucose-6-phosphatase ↓Aspartate transaminase ↓Alanine transaminase ↓Acid phosphatase ↓Alkaline phosphatase	(90)
Root methanolic extracts of *W. somnifera* (200 mg/kg/day), IP, for eight weeks	Streptozotocin-induced diabetes in male Wistar rats, two weeks before receiving *W. somnifera*	↓Blood glucose ↓Serum aspartate ↓transaminase ↓Alanine transaminase ↓Alkaline phosphatase ↓Total cholesterol ↓TG ↓LDL-c ↑Albumin: globulin ratio	(91)
Methanolic root extract of *W. somnifera* (25, 50, 100, 200, or 400 mg/kg/day), orally, for 10 days	-Streptozotocin and nicotinamide induced diabetes in male albino mice, a week before *W. somnifera *therapy -foot shock stress-triggered hyperthermia test during *W. somnifera*	↓Body weight ↓Basal core temperature ↓Stress-triggered hyperthermia ↓Plasma glucose levels ↑Plasma insulin levels ↓ Plasma cortisol levels ↓Adrenal gland/ body weight ↓Spleen/ body weight	(92)
Withaferin-A (2 & 10 mg/kg (dose interval indeterminate)), IP, for 28 days	Streptozotocin-induced diabetes in Swiss albino mice, in the first five days of receiving Withaferin-A	↓Blood glucose levels ↑Glucose tolerance test ↓Pancreatic MDA level ↑Pancreatic glutathione level ↓Pancreatic nitrite level ↑Plasma insulin ↓TNF-α ↓IL-6 ↓NFκB ↑Nrf2 ↓Apoptosis ↓Pancreatic insults	(93)
Aqueous root extract of *W. somnifera *(100, 200, and 400 mg/kg/day), orally, for 5 weeks	Streptozotocin-induced diabetes in 2-day-old Wistar rat pups, 19 days before *W. somnifera*	↑Superoxide dismutase ↑Catalase ↓Blood glucose ↓MDA ↑GSH ↑Glutathione peroxidase ↑Glutathione-S-transferase ↑Glutathione reductase ↓Fibrosis of pancreatic acini with regeneration of β-cells	(95)
*W. somnifera* root hydroalcoholic extract (dose, duration of therapy, and the way of administration indeterminate)	Corticosteroid and high sucrose diet-Induced type 2 diabetes in Wistar rat (sex indeterminate)	↓HbA1c ↓Insulin ↓Glucose levels ↓Insulin resistance ↓β-cell dysfunction) ↓Total cholesterol ↑HDL ↓TG ↓LDL-c ↓VLDL ↓Free fatty acid ↓Degenerative change in the pancreatic ↑Glutathione peroxidase ↑Superoxide dismutase ↑Catalase ↑GSH	(96)
*W. somnifera* root hydroalcoholic extract	*In vitro* assay	Inhibition of DPP-4 up to 77.3 %	
Withaferin – A	*In silico* analysis	the highest binding affinity with DPP-4	
Root powder of *W. somnifera *(1000 mg three times a day), orally, 30 days	Non-insulin-dependent diabetes mellitus human subjects	↓Blood glucose ↑Urine sodium ↑Urine volume ↓Serum cholesterol ↓TG ↓LDL ↓VLDL	(103)
Dianex (a poly herbal formulation consisting of the aqueous extracts of *Gymnema sylvestre, Eugenia jambolana, Momordica charantia, Azadirachta indica, Cassia auriculata, Aegle marmelose*, *Withania somnifera*, and *Curcuma longa*); (100, 250, and 500 mg/kg/day), orally, in acute (6h) and long-term (6 weeks)	Streptozotocin-induced diabetes in Swiss albino mice (either sex), a week before Dianex treatment	↓Blood glucose ↑Oral glucose tolerance ↓Total cholesterol ↓HDL ↓TG ↑Liver glycogen ↑Liver protein ↓Inflammation, necrosis, and degeneration of pancreas and liver tissues	(102)

ATGL: Adipose triglyceride lipase; C/EBP: CCAAT enhancer binding protein β; CD36: cluster of differentiation 36; CPT1: carnitine palmitoyltransferase 1; DPP-4: ipeptidyl peptidase 4; FAS: fatty acid synthase; GCK: glucokinase; GSH: Glutathione; GLUT4: Glucose transporter 4; HbA1C: A hemoglobin A1C; HDL: high-density lipoprotein, HSL: Hormone sensitive lipase; IL-6: Interleukin-6; LDL: low-density lipoprotein, MDA: Malondialdehyde; NFκB: Nuclear factor kappa-light-chain-enhancer of activated β-cells; Nrf2: Nuclear factor erythroid 2-related factor 2; PCK1: phosphoenolpyruvate carboxykinase; PGC1α PPARγ coactivator 1α; PPARγ peroxisome proliferator-activated receptor gamma; PPAR-: Peroxisome proliferator-activated receptor-; RIN-5F: rat pancreatic β-cell line derived from an insulinoma, SREBP-: sterol regulatory element binding protein; TG: triglyceride, TNFα: Tumour necrosis factor α; UCP1: Uncoupling protein 1, VLDL: very-low-density lipoprotein

This schematic illustration outlines the various mechanisms by which *W. somnifera* (commonly known as Indian ginseng) exerts its antidiabetic effects. *W. somnifera* inhibits DPP-4, reduces the conversion of polysaccharides to glucose, and increases glucose uptake; furthermore, it mitigates oxidative stress and inflammation and may decrease the expression of PPAR-γ in the liver and adipocytes.. The red symbols indicate the effects of Indian ginseng and its secondary metabolites, with "↑" representing promotion or activation and "↓" indicating inhibitory or suppressive effects.

Activating sign:

Blocking sign:

This schematic illustration outlines the various mechanisms through which *W. somnifera* (commonly known as Indian ginseng) exhibits antihypertensive activity. *W. somnifera* has diuretic and vasodilatory effects, helping to lower blood pressure. Additionally, it plays a role in modulating oxidative stress and inflammation. The downward arrow “↓” indicates the inhibitory or suppressive effects of Indian ginseng and its secondary metabolites.

**Table 2:** Effects of Indian ginseng or its constituents on hypertension, dyslipidemia, and atherosclerosis

Condition	Indian ginseng or its constituents	Experimental model	Results	Ref.
Hypertension	Methanol root extract of *W. somnifera* (50 and 100 mg/kg/day), orally, for 35 days	Monocrotaline-induced pulmonary hypertension in male Sprague–Dawley rats, accompanied by *W. somnifera *therapy, or 21 days before *W. somnifera*	↓Body weight ↓Right ventricular pressure ↓Right ventricular hypertrophy ↓Pulmonary vascular remodeling ↑Procaspase-3 in the lung tissue ↓Reactive oxygen species in the lung tissue ↑IL-10 in lungs ↓TNF-α in the lung tissue ↓NFkB expression in the lung tissue ↑Vasorelaxation in pulmonary vessels ↑Endothelial nitric oxide synthase expression in the lung tissue ↓Hypoxia-inducible factor 1-alpha expression in the lung tissue	(106)
Dyslipidemia and atherosclerosis	Isolated Withasilolide F, E, Withasomniferol B, Withasilolides G, H, and I from methanolic root extract of *W. somnifera* (12.5 and 25 mM) for 24 hr	3T3-L1 preadipocytes	Significantly inhibited adipogenesis suppressed the enlargement of lipid droplets ↓ mRNA expression levels of Fabp4 and Adipsin (the adipocyte marker genes) suppress adipogenesis in the 3T3-L1 preadipocytes ↑ mRNA expression of HSL and ATGL, lipolytic genes in the 3T3-L1 cells ↑Lipid metabolism by promoting lipolysis ↓Lipogenesis	(113)
	Aqueous root extract of *W. somnifera *(200 and 400 mg/kg/day) for 5 weeks, orally	Streptozotocin induced diabetes in 2-day-old newborn rat pups (species indeterminate), 90 days before receiving *W. somnifera*	↓Blood glucose ↓MDA ↑Reduced glutathione ↓Total cholesterol ↑HDL ↓TG ↓LDL-c ↓VLDL ↓Lactate dehydrogenase ↓Creatinine kinase ↑Glutathione peroxidase ↑Glutathione reductase ↑Glutathione-S-transferase ↑Superoxide dismutase ↑Catalase	(116)
	Ethanolic root extract of *W. somnifera *(250, 500, and 1000 mg/kg/day), orally, for 28 days	Nicotinamide and streptozotocin-induced diabetes in Wistar rats (both sex*es)*	↓Total cholesterol, LDL ↑HDL levels	(117)
	Root powder of *W. somnifera* (0.75 and 1.5 g/day), orally, for 4 weeks	Hypercholesteremic Charles Foster male rats (induced by the addition of 0.5 % cholesterol and 1% sodium taurocholate to the standard Commercial diet), accompanied by the *W. somnifera* therapy	↓Total lipid ↓Total cholesterol ↑HDL ↓TG ↓LDL ↓VLDL ↓Atherogenic index ↓HMG-CoA reductase ↑Bile acid ↑Fecal cholesterol ↑Fecal bile acid ↑Neutral sterol	(119)
	Ethanol root extract of *W. somnifera *(50 and 100 mg/kg, (dose interval indeterminate)), orally for six weeks	High cholesterol diet in male rabbits, for six weeks, accompanied by the *W. somnifera* therapy	↓Total cholesterol ↑HDL ↓TG ↓LDL-c ↓VLDL ↓MDA ↑GSH	(120)

GSH: Glutathione; HDL: high-density lipoprotein; HMG-CoA: 3-hydroxy-3-methylglutaryl coenzyme A; IL-10: Interleukin-10; LDL: low-density lipoprotein; MDA: Malondialdehyde; NFκB: Nuclear factor kappa-light-chain-enhancer of activated β-cells; TG: triglyceride; TNFα: Tumour necrosis factor α, VLDL: very-low-density lipoprotein

The schematic illustration outlines various mechanisms through which *W. somnifera *exerts its antihyperlipidemic and antiatherosclerotic effects. Specifically, *W. somnifera* inhibits the activity of HMG-CoA reductase, which is essential for cholesterol synthesis, and increases the production of bile acids. They regulate the gene expression of SREBP-1c, PPAR-γ, and CYP7A1 and reduce inflammation, oxidative stress, and thrombin activity.

HMG-CoA: 3-hydroxy-3-methylglutaryl coenzyme A, PPAR-: peroxisome proliferative receptor; SREBP: Sterol Regulatory Element-Binding Protein.

The red indicator highlights the effects of Indian ginseng and its secondary metabolites: ↑ indicates promotion or activation, while ↓ signifies inhibitory or suppressive effects.

Activating sign:

## Declaration

Vector icons were obtained through smart.servier.com and vecteezy.com. During the preparation of this work, the authors used AI-assisted technologies (Grammarly and iThenticate) to rephrase, reduce plagiarism, and improve language and grammar. After using this tool/service, the authors reviewed and edited the content as needed and take full responsibility for the content of the publication.
